# SDIMMMER: A Proposed Clinical Approach to Optimize Cellular Physiology in Regenerative Medicine

**DOI:** 10.3390/life14101287

**Published:** 2024-10-11

**Authors:** João Vitor Lana, José Fábio Lana, Gregory Melo, Gabriel Ohana Marques Azzini, Gabriel Silva Santos, Tomas Mosaner, Daniel de Moraes Ferreira Jorge, Lucas Furtado da Fonseca, André Kruel, Fábio Ramos Costa, Madhan Jeyaraman, Alex Pontes de Macedo, Napoliane Santos, Luyddy Pires, Claudia Herrera Tambeli

**Affiliations:** 1 Medical School, Max Planck University Center (UniMAX), Indaiatuba 13343-060, SP, Brazil; jvblana@gmail.com (J.V.L.); josefabiolana@gmail.com (J.F.L.); gregory.junior599@al.unieduk.com.br (G.M.); 2 Department of Orthopedics, Brazilian Institute of Regenerative Medicine (BIRM), Indaiatuba 13334-170, SP, Brazil; drgabriel.azzini@gmail.com (G.O.M.A.); tmosaner@uol.com.br (T.M.); danfjorge@gmail.com (D.d.M.F.J.); ffonsecalu@gmail.com (L.F.d.F.); kruel.andre@gmail.com (A.K.); alex_macedo@icloud.com (A.P.d.M.); dranapolianesantos@gmail.com (N.S.); luyddypires@gmail.com (L.P.); 3 Regenerative Medicine, Orthoregen International Course, Indaiatuba 13334-170, SP, Brazil; fabiocosta123@uol.com.br (F.R.C.); madhanjeyaraman@gmail.com (M.J.); 4 Clinical Research, Anna Vitória Lana Institute (IAVL), Indaiatuba 13334-170, SP, Brazil; 5 Medical School, Jaguariúna University Center (UniFAJ), Jaguariúna 13820-000, SP, Brazil; 6 Department of Orthopedics, FC Sports Traumatology, Salvador 40296-210, BA, Brazil; 7 Department of Orthopedics, ACS Medical College and Hospital, Dr. MGR Educational and Research Institute, Chennai 600077, Tamil Nadu, India; 8 Institute of Biology, State University of Campinas (UNICAMP), Campinas 13083-852, SP, Brazil; tambeli@unicamp.br

**Keywords:** metabolism, systemic inflammation, clinical diagnosis, regenerative medicine

## Abstract

SDIMMMER is an acronym intended for use in both clinical practice and medical research. It facilitates a comprehensive evaluation of a patient’s metabolic profile and serves as a mnemonic for the following key assessment areas: Sleep, Diet, Microbiome, Metabolism, Medications, Exams, and Rehabilitation. In the clinical setting, SDIMMMER’s primary objective is to monitor and manage the patient’s metabolic status, particularly targeting low-grade chronic systemic inflammation, a hallmark of metabolic syndrome (MS). This inflammatory condition is characterized by elevated levels of circulating inflammatory cytokines and increased macrophage infiltration in peripheral tissues. SDIMMMER aims to enhance the effectiveness of ortho biological treatments by elevating growth factor levels, thereby enhancing patient outcomes. Additionally, SDIMMMER emphasizes guiding patients toward positive lifestyle changes to improve overall quality of life and foster a healthier metabolism. SDIMMMER introduces a patient metabolic profile quantification tool comprising 7 domains, totaling 35 items. Additionally, an instructional guide is provided to facilitate the application process. Its versatility spans various clinical and research domains, showcasing its potential to positively influence multiple fields.

## 1. Introduction

The success of regenerative therapies hinges on many factors influencing the patient’s overall health and response to treatment. Sleep patterns, dietary habits, microbiome composition, metabolic status, medication usage, and the patient’s general health condition play pivotal roles among these factors. Numerous studies have highlighted the direct impact of these factors on the efficacy and outcome of regenerative interventions [1,2,3]. Therefore, a systematic and quantitative assessment of these parameters is imperative to mitigate biases in clinical research and optimize the outcomes of regenerative therapies. This necessitates the development and implementation of comprehensive evaluation tools that can accurately measure these multifaceted variables, thereby enhancing the efficacy and reproducibility of regenerative medicine interventions.

**Figure 1:**
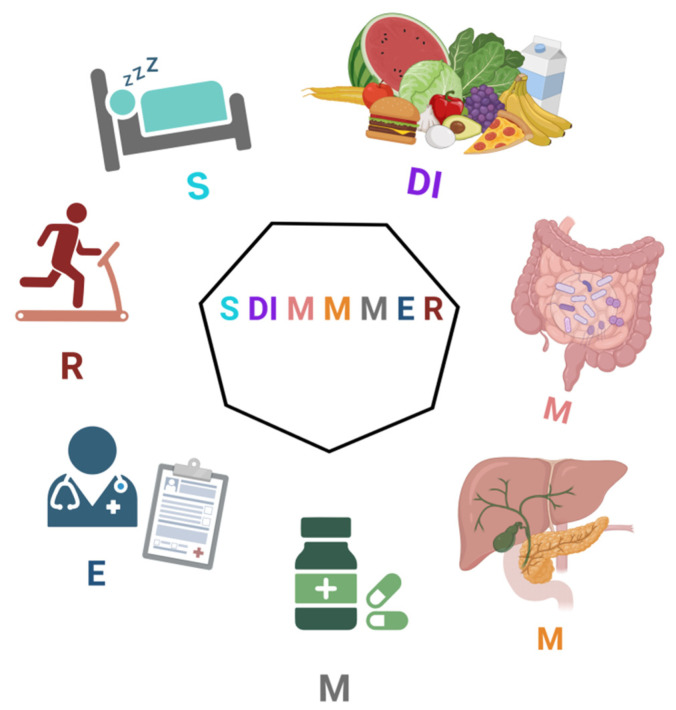
SDIMMMER Acronym.

Therefore, the aim of this article is to propose the acronym “SDIMMMER” (Figure 1), which encompasses the critical aspects of patient assessment: Sleep, Diet, Microbiome, Metabolism, Medications, Exams, and Rehabilitation. This acronym devised by Dr. José Fábio Lana, presents a patient metabolic profile quantification tool with 7 domains, encompassing 35 items. Furthermore, an instructional guide is included to streamline the application process.

“SDIMMMER” is both a research tool and a novel propaedeutic approach intended to assist physicians in comprehensively evaluating a patient’s metabolic profile and devising tailored strategies to enhance the precision of clinical diagnosis and increase the treatment success rate. This innovative research tool and set of propaedeutics represents a pioneering technique in regenerative medicine. The primary objective of SDIMMMER is to meticulously monitor the patient’s metabolic status and mitigate low-grade chronic systemic inflammation, commonly observed in patients with metabolic syndrome (MS) [4]. This inflammatory state is characterized by high levels of circulating inflammatory cytokines and increased macrophage infiltration in peripheral tissues [5].

Importantly, unlike acute inflammation, low-grade systemic inflammation does not directly cause tissue damage or loss of function, distinguishing it as a unique feature [6]. The term ‘meta-inflammation’ has been used to describe this phenomenon, highlighting its intimate association with the onset of cardio-metabolic diseases [7].

## 2. Meta-Inflammation

**Figure 2:**
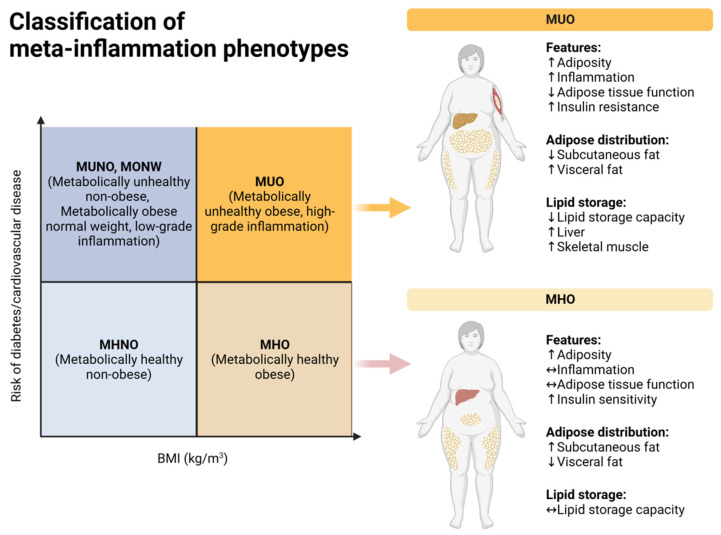
Classification of meta-inflammation phenotypes. Up arrows means increased (more), and down arrows means decreased (less). Sideway arrows indicate equilibrium.

By employing our proposed acronym, researchers and physicians can more effectively manage meta-inflammation in patients while correlating with lifestyle habits. Frequently, issues related to sleep, diet, gut microbiome, metabolism, medications and their interactions, along with poor clinical diagnosis and rehabilitation, may manifest in many patients with various chronic conditions. Within most medical practices, patients may exhibit or not have an evident low-grade systemic inflammation profile, which often correlates strongly with symptom complaints. Patients with altered BMI or a history of conditions like hypertension and diabetes frequently exhibit a “high-grade” systemic inflammation profile [1,2,3]. In the context of regenerative medicine, the concept of “inflammation profiles”, can be delineated into “low-grade” and high-grade” states of meta-inflammation [8,9], as depicted in Figure 2.

The concept of meta-inflammation is linked to a patient’s lifestyle, and physical attributes such as a distended abdomen, accumulation of visceral fat tissue, or allergic tendencies. Consequently, our focus extends beyond treating diseases alone to addressing the overall well-being of patients. A high inflammatory grade or likelihood of escalation predisposes patients to develop complex metabolic disorders and additional complications [7,8,9,10,11].

Hence, medical interviews should ideally commence with a thorough investigation and maintain a strict follow-up of clinical evolution. Encouraging healthy lifestyle habits, evaluating clinical exams, and implementing rehabilitation protocols are key steps aimed at regulating metabolic processes.

## 3. Components of SDIMMMER

### 3.1. Sleep

In regenerative medicine, evaluating the quality of sleep is a primary factor in examining patients with meta-inflammation symptoms. Table 1 lists typical strategies aimed at improving sleep quality. These strategies are beneficial for an initial assessment of sleep in patients. However, if individuals show extremely poor sleep patterns, physicians can utilize tools such as the Pittsburgh Sleep Quality Index (PSQI), Mini-Sleep Questionnaire (MSQ), and Jenkins Sleep Scale (JSS), among others [12].

Explanatory Notes:

Sleep and Metabolism: Poor sleep is linked to impaired glucose metabolism and increased cortisol levels, which affect energy balance and inflammation.

Sleep and Diet: Disrupted sleep patterns can increase cravings for high-fat, high-sugar foods, negatively impacting the microbiome and metabolic health.

Sleep and Rehabilitation: Adequate rest enhances muscle recovery and tissue repair, which are crucial in the rehabilitation process.

Sleep and Microbiome: Circadian rhythm disruptions can alter gut microbiota composition, increasing systemic inflammation and metabolic disturbances.

These tools help map out sleep processes in patients with metabolic disorders, considering the physiological impact of sleep on the development and progression of these conditions. Conversely, poor sleep plays a fundamental role in the hormonal regulation of cortisol and growth hormone (GH) [13,14]. Elevated cortisol levels in the bloodstream induce metabolic stress, subsequently leading to oxidative stress. Sleep deprivation studies reveal an increase in proinflammatory cytokines, altering the immune response and releasing biomarkers such as interleukin-6, TNF-alpha and C-reactive protein [15].

Sleep also regulates appetite by influencing leptin and ghrelin, two major hormones. Leptin, produced by the adipose tissue, suppresses appetite, whereas ghrelin, released in the stomach, stimulates hunger. Leptin levels are relieved to rise during sleep due to melatonin’s influence. Insulin-triggered leptin production inhibits ghrelin, ending the feeling of hunger during sleep [15,16]. Sleep deprivation studies show an imbalance in the leptin–ghrelin ratio in individuals sleeping only for four hours per night. One study found an 18% reduction in leptin and a 28% increase in ghrelin in sleep-deprived individuals compared to controls. Thus, focusing on patients’ sleep quality as part of an examination can help regulate appetite and food metabolism. This strategy can improve dietary habits and support weight loss for BMI adjustments [15]. Sleep is integral to the secretion of excitatory neuropeptides called orexins, produced by neurons in the perifornical region of the hypothalamus. Orexins are associated with energy homeostasis, balancing calorie intake and energy expenditure, and influencing the hypothalamus to regulate this process. Leptin, ghrelin and glucose are linked to orexins, providing physicians with valuable insights into sleep and metabolism [16,17,18]. Recently, a hypothesis proposing the therapeutic role of orexin has emerged. A study on glucose metabolism in knockout mice showed that those lacking orexin-coding genes had significantly altered insulin sensitivity and weight gain [19]. Adequate sleep plays a crucial role in tissue repair, immune regulation, and hormone balance [20]. During sleep, the body releases growth hormones that are essential for cell regeneration and repair [21]. In regenerative medicine, poor sleep can slow healing processes and increase systemic inflammation, whereas optimizing sleep can enhance the body’s natural regenerative capacity.

### 3.2. Diet—We Are What We Eat

Dietary habits significantly influence the phenotype of phagocytic bacteria, such as commensalists [22,23]. The growth of the gut microbial population may vary with age. Depending on the predominance of specific bacterial phenotypes, proinflammatory cytokines may be released. When these cytokines become prevalent in the human gastrointestinal tract (GIT), elevated concentrations can establish a state of chronic low-grade inflammation. Prolonged low-grade inflammation can cause the microbial population to remain imbalanced, leading to dysbiotic shifts [9,24,25,26]. Dysbiosis impairs nutrient absorption and disrupts the secretory activity of gut-resident bacteria, which are directly linked to various pathologies, including neuropsychiatric disorders [27]. These pathophysiological processes are caused by the fragilization of the mucin barrier in the GIT. High levels of proinflammatory molecules place the intestinal tract under significant oxidative stress, initiating the oxidation of glycoproteins, decomposing the mucin barrier, and exposing native intestinal structures. When the gut is exposed, proinflammatory cytokines extravasate into the bloodstream, increasing the host’s susceptibility to injuries and diseases, a condition known as leaky gut syndrome [25,28,29,30].

Therefore, an individual’s microbiome is a reflection of their dietary habits. Unhealthy foods shape the microbiome and favor the growth of commensal bacteria [31]. The elevated growth of commensalists leads to excessive production of proinflammatory cytokines, exacerbating oxidative stress and the proinflammatory role of immune cells. This kind of systemic inflammation is present in MS, making it a crucial factor to evaluate in patients. It is reasonable to deduce that individuals with poor dietary habits will have a predominantly poor microbiome, resulting in metabolic problems and disrupted tissue homeostasis [31,32]. A balanced diet rich in anti-inflammatory foods (such as omega-3 fatty acids, fruits, and vegetables) can help reduce chronic inflammation, which is known to impair tissue regeneration [33]. In regenerative medicine, a diet that supports a healthy metabolism can improve nutrient supply to tissues, promoting better outcomes in healing and cellular repair. For these reasons, physicians must understand patient metabolism and delve deeper into their history. This includes gathering information such as: family history of diseases; childhood trauma; substance abuse (including alcohol, tobacco, marijuana, opioids, and other drugs), sexual activity, vaccination records, and level of physical activity.

### 3.3. Microbiome

Systemic low-grade inflammation in MS is influenced by various factors, including sleep disturbances and imbalanced circadian rhythms. To better understand systemic inflammation and MS, it is essential to analyze diet and gut microbiome composition. Evaluation of dietary habits allows physicians to hypothesize about the gut microbiota based on a patient’s daily meals. A poor microbiome is often associated with the consumption of refined carbohydrates, wheat and milk products, and insufficient dietary fibers and lean proteins. The gut microbiome encompasses a diverse microbial population, including bacteria, fungi and even some viruses, which must be adequately balanced to maintain symbiotic homeostasis between the gastrointestinal cells and gut flora.

GI tract homeostasis is crucial for human health, as it is intrinsically linked to intestinal microbiota composition. Approximately 90% of the human intestinal microbiome consists of Firmicutes and Bacteroidetes, which play a role in fermentating non-starch polysaccharides (NSP) and synthesing short-chain fatty acids (SCFA) [34,35]. This fermentation process significantly contributes to the production of mucin, a glycoprotein that serves as a barrier against microbial invasion. Over time, bacterial populations are renewed through selection based on the nutrients they receive [28]. A balanced diet, with the inclusion of prebiotics and probiotics, is a key determinant of gut flora composition [31]. Conversely, a diet high in wheat, milk, sugar, gluten and refined carbohydrates combined with low quantities of fibers, vegetables, and lean proteins, leads to poor equilibrium of the microbiota. Optimizing microbiome health through dietary modifications or probiotics can lead to reduced chronic inflammation and improved metabolic functioning, contributing to faster recovery and better tissue health.

### 3.4. Metabolism

The treatment aims to manage metabolism in MS patients affected by chronic low-grade systemic inflammation, targeting high-standard parameters and solid biomarker results. This approach is based on adopting physiological treatments for the inflamed organism, with the primary objective of establishing homeostasis in a dysregulated metabolism by guiding patients to adopt healthier daily habits. These include adhering to healthy diets such as the Mediterranean diet, addressing sleep disturbances, encouraging physical activity, and gradually gaining control and correction of the disordered metabolism. Collectively, these strategies contribute to reducing systemic low-grade inflammation. A healthy metabolic state is essential for maintaining energy balance and efficient cellular repair mechanisms. Metabolic dysfunction, such as insulin resistance, can impair the body’s ability to regenerate tissues. By improving metabolic health, patients can experience an enhanced energy supply to cells, which is vital for successful outcomes in regenerative medicine.

The interaction of medications and supplements aids in recovering metabolism from chronic inflammatory stimuli, helping the organism transition from a senescent state to a functional state. In this conceptual framework, a functional and efficient metabolism is imperative for harvesting healthy cells, whether from bone marrow-derived products or growth factors from peripheral blood. The goal is to utilize the best and richest biological products containing growth factors and monoclonal cells. When the metabolism is non-inflamed and well-regulated, the body produces blood rich in pro-healing factors. Therefore, by guiding and monitoring the metabolic aspects of patients, physicians can anticipate improved levels of growth factors and monoclonal cells. This, in turn, is expected to yield better treatment outcomes and feedback [36,37,38].

#### Feasibility of Evaluating Metabolism and Microbiome in Clinical Practice

The SDIMMMER framework emphasizes the importance of evaluating both metabolism and microbiome comprehensively. While these components may initially seem challenging to assess in routine clinical practice, several practical tools and strategies can be implemented effectively.

Metabolism Evaluation:

Metabolic health can be readily assessed in most clinical settings using standard tests. Blood tests, such as fasting glucose, insulin levels, and lipid profiles, are commonly used to evaluate a patient’s metabolic state. These tests provide insight into insulin sensitivity, cholesterol levels, and the risk of metabolic syndrome. Body composition analyses, such as BMI, waist-to-hip ratio, and bioelectrical impedance analysis (BIA), are also useful and non-invasive ways to evaluate metabolic health.

Microbiome Evaluation:

Comprehensive microbiome evaluation may be more specialized, but clinicians can begin with dietary and symptom questionnaires. These tools assess gut health indicators like fiber intake, probiotic consumption, and digestive symptoms (e.g., bloating, constipation). For deeper analysis, non-invasive stool tests, such as 16S rRNA sequencing, are becoming more accessible and can provide detailed insights into microbiome health when necessary.

Stepwise Approach:

We propose a stepwise approach for metabolism and microbiome assessment within the SDIMMMER framework. Clinicians can start with routine assessments like blood tests and dietary evaluations and escalate to specialized tests if initial findings suggest a need for more detailed analysis. Collaboration with specialists in gastroenterology or endocrinology may be warranted in complex cases.

### 3.5. Medications

It is crucial to evaluate medications and their potential interactions, considering not only interactions between medications but also with the patient’s lifestyle habits. Food-drug and alcohol–drug interactions should be suspected when a patient adheres to their medication regimen correctly, yet the therapy remains inefficient or adverse effects increase. In such scenarios, a careful evaluation of dietary and prescription history is recommended to identify possible food–drug interaction [39]. Common drug interactions occur with fruits (especially grapefruit), dairy products, vitamin K, foods containing tyramine, and alcohol [39].

Drug–drug interactions occur when one drug alters the effect of another, known as pharmacodynamic interaction, or affects its metabolism and distribution, known as pharmacokinetic interaction [39]. Many common drug–drug interactions involve cytochrome P450 enzymes [40]. Other interactions arise from additive effects, such as combinations of drugs that increase the risk of seizures, prolong the QT interval, increase central nervous system depression, and raise the risk of serotonin syndrome [41,42]. Regenerative physicians can enhance their awareness and optimize drug safety by using drug interaction software [43].

A study showed that potential drug–drug interactions were associated with 10.8% of 276,891 community pharmacy prescriptions [44]. The study found that most potentially serious interactions in outpatients involve a limited number of drugs. Methotrexate and warfarin had the highest risk of causing potentially serious (class D) interactions, particularly with NSAIDs. NSAIDs were the most commonly interacting drugs in this study.

It is also vital to investigate the use of anticoagulants and platelet inhibitory agents, as they can increase the risk for abnormal bleeding [45,46]. This is particularly important for patients undergoing regenerative procedures with orthobiologics, where platelets play a crucial role [47]. Medications such as adenosine diphosphate receptor antagonists (clopidogrel and prasugrel), acetylsalicylic acid (aspirin), and integrin αIIbβ3 (GPIIb-IIIa) receptor blockers (abciximab, eptifibatide, and tirofiban) are well-known antiplatelet drugs [48]. Other commonly used agents, including nonsteroidal anti-inflammatory drugs, antibiotics, cardiovascular and lipid-lowering drugs (statins) and selective serotonin reuptake inhibitors (SSRI) can also impair platelet function and interfere in certain clinical settings [48].

In regards to diabetes, metformin has indeed been used to effectively reduce blood glucose levels; however, it also reduces circulating levels of vitamin B12, an essential element of regeneration [49]. Vitamin B12 is involved in DNA synthesis, red blood cell formation and neurological function [50]. Its deficiency results in megaloblastic anaemia, peripheral neuropathy and, potentially, a reduced regenerative capacity of cells [50]. Metformin interferes with vitamin B12 absorption by reducing the absorption of the vitamin by intrinsic factor, a glycoprotein important for its absorption in the ileum [51].

The use of proton pump inhibitors (PPI) is also another aspect that requires careful monitoring. A recent study reported that prolonged use of PPIs can impair the absorption of dietary iron, vitamin B12, and proteins, which are crucial for cellular renewal [52]. Iron is an essential component for the production of haemoglobin, the protein that carries oxygen from the lungs to the rest of the body, and thus plays a key role in our oxygen transport system [53]. In addition to our red blood cells, iron is present in many enzymes, most of which are required for the metabolism of biological molecules in our bodies [54]. Vitamin B12, on the other hand, besides the formation of red blood cells, is also important for proper neurological function. PPIs cause a rise in gastric pH by reducing the acidity in the stomach, leading to a decrease in non-heme iron and vitamin B12 absorption [55].

Furthermore, PPIs are known to also have an effect on pepsinogen activation. The digestive enzyme, pepsin, is produced by the conversion of pepsinogen (an inactive precursor) in gastric acid. PPIs suppress gastric production of acid, and therefore inhibit the conversion of pepsinogen to pepsin. This may hinder protein digestion and absorption [56]. Lack of protein absorption may lead to a deficiency of essential amino acids necessary for cellular regeneration and tissue repair [57].

Optimizing medication use, and minimizing unnecessary or harmful medications, helps to reduce adverse effects on metabolism, immune function, and cellular repair, thus supporting better outcomes. For these reasons, patient counseling and collaboration between regenerative medicine physicians are essential to help patients avoid drug interactions and enhance the effectiveness of regenerative treatments.

### 3.6. Exams

Regular physical examinations allow for early detection of inflammation, tissue damage, or functional deficits that may affect regenerative outcomes. By monitoring these signs, clinicians can intervene earlier to optimize the regenerative process through timely interventions like physical therapy or regenerative treatments. To better correlate the patient’s metabolic health status with the information obtained during medical interviews, a comprehensive blood check-up is beneficial. This includes a complete blood count and secondary laboratory analyses to measure serum concentrations of key biomarkers such as albumin, zinc, C and D vitamins, C Reactive Protein, ferritin, homocysteine, TSH, free T3 and T4, Testosterone, DHEA, and alkaline phosphatase. Table 2 provides detailed insights into these biomarkers and their clinical relevance. These analyses offer practitioners a broader perspective on the patient’s metabolic health, enabling the design of more precise therapeutic interventions. After analyzing the results, the next step is to regulate the concentrations of these biomarkers appropriately and reevaluate previous medication interactions if the patient has already started pharmacological treatment for a specific condition. Physicians must be mindful of all prescribed medications and initiate the treatment accordingly.

### 3.7. Rehabilitation

Rehabilitation is an essential component of the SDIMMMER framework, as it directly influences a patient’s recovery and long-term health outcomes. By incorporating structured physical therapy and personalized rehabilitation programs, clinicians can promote tissue regeneration, reduce chronic inflammation, and enhance cellular physiology [69]. Rehabilitation plays a key role in addressing musculoskeletal disorders and other chronic conditions, particularly when combined with regenerative medicine therapies [70]. According to a recent case report [71], a 47-year-old female with bilateral knee osteoarthritis presented with chronic pain and difficulty in performing daily activities, such as walking and standing. She underwent a six-week rehabilitation program combining recent physiotherapy techniques, including Mulligan mobilisation and Kinesio taping, along with conventional methods like strength training and joint mobilization. Pain management was achieved through hot packs and interferential therapy (IFT), while targeted exercises for the quadriceps and hamstrings, along with balance training, improved knee stability and function. Mulligan mobilisation helped reduce joint pain and enhance alignment, and Kinesio taping provided additional support for joint stability. By the end of the program, the patient experienced significant pain reduction (VAS scores decreased from 5/10 to 1/10), improved knee mobility (an increase of 10° in knee flexion), and enhanced functional ability, as reflected by an improvement in the WOMAC score from 29/100 to 10/100. This case illustrates how a combined rehabilitation approach can effectively improve pain, function, and mobility in osteoarthritis patients, aligning with the goals of the SDIMMMER framework.

Moderate physical exercise is associated with a decreased risk of arthritis, suggesting a protective effect against cartilage degradation [72]. It is essential to propose an exercise program that combines endurance and strength for patients before and after regenerative procedures. Movement around arthritic joints and diseased tendons has been shown to increase functional capacity and reduce pain [73]. Exercise can dramatically alter strain sensing, extracellular matrix composition, and inflammation, likely influencing the quantity and function of stem cells post-exercise [74].

For lower limb rehabilitation, we recommend the following four-phase program:

Immediately Post-Intervention (Day 0–3): Focus on protecting the treated area and controlling pain as needed. Encourage range of motion within tolerance and improve joint mobility. Patient education about the phases of regeneration is vital during these initial days.

Day 3–14 Post-Treatment: Increase local circulation and tissue tolerance through exercise. Enhance muscular strength and endurance, progress to full range of motion, and avoid deconditioning. Continue patient education about weight management and advise against deep squatting, twisting, lunges, and deep knee and hip flexion activities. Encourage isometric exercises such as bridges and knee extensions.

Two to Four Weeks Post-Treatment: Progress in muscular strength and endurance training. Introduce open and closed chain exercises, balance, and proprioception training on stable surfaces, and initiate aquatic therapy.

Five to Twelve Weeks Post-Treatment: Achieve full muscular strength recovery and progress to balance and proprioception training on unstable surfaces. Gradually increase low-impact activities and weights. Encourage full-range movements with heavier loads and allow activities such as cycling, brisk walking, and hiking as tolerated.

For the upper body and upper limbs, similar phases are followed. Although the quantitative and qualitative parameters of the mechanical forces cannot yet be controlled at the cellular level, partial control at the macroscopic level is achievable. Thus, movement is paramount for the optimization of any regenerative procedure [69].

#### Author’s Note

For further clarification, we created a grading scale (Table 3) for the SDIMMMER approach. This table provides an overall score for the patient based on questions for each component of the acronym. The total score equals 140 (7 domains, with a maximum of 20 points per component). Therefore, the closer the patient is to this number, the better their cellular health and physiology.

## 4. Limitations

While the SDIMMMER framework offers a comprehensive approach to assessing and optimizing key metabolic and physiological domains, there are several limitations that should be acknowledged.

Subjectivity of Patient-Reported Data: Components such as Sleep and Diet rely heavily on self-reported information, which can be subjective and prone to inaccuracies. For example, patients may under—or overestimate their sleep duration or misreport dietary habits, which can affect the accuracy of the assessment.

Variability in Microbiome Composition: The gut microbiome is highly individual and influenced by numerous factors, including genetics, environment, and lifestyle. This variability may make it difficult to draw consistent conclusions across different patient populations, limiting the generalizability of the findings.

Complexity of Implementation: The comprehensive nature of the SDIMMMER framework may pose challenges in clinical practice, particularly in time-constrained settings. Incorporating detailed assessments of Sleep, Diet, Microbiome, and Metabolism requires interdisciplinary collaboration, which may not always be feasible in all healthcare settings.

Lack of Empirical Validation: As the SDIMMMER framework is still in its developmental stage, there are no clinical trial data currently available to validate its effectiveness. Future studies will be necessary to empirically test the framework’s utility and optimize its application in various clinical contexts.

## 5. Conclusions

We introduce and propose the “SDIMMMER” acronym, crafted by Dr. José Fábio Lana, as a transformative propaedeutic approach in regenerative medicine. This methodology emphasizes key aspects of a patient’s life: Sleep, Diet, Microbiome, Metabolism, Medications, Exams, and Rehabilitation. The goal is to improve the precision of clinical diagnoses and elevate treatment success rates.

The intricate interplay between these factors becomes apparent when considering the correlation with systemic inflammation, particularly in individuals with metabolic syndrome. The SDIMMMER approach delves into the root causes of chronic inflammation, highlighting the significant impact of daily habits on overall health.

The prevalence of issues related to SDIMMMER across various diseases underscores the universal nature of these problems. By tailoring a holistic approach and addressing core lifestyle aspects, the objective is not only to enhance metabolic function but also to pave the way for healthier cells. This comprehensive strategy augments the efficacy of orthobiological treatments, resulting in improved patient outcomes and prioritizes the long-term well-being of individuals even after they receive the treatment they need.

Ultimately, the approach we propose is a theoretical framework designed to optimize cellular physiology in regenerative medicine by addressing key metabolic domains. At this stage, the SDIMMMER methodology has not yet been applied in clinical settings. Future studies and clinical trials will be conducted to validate the effectiveness of the SDIMMMER framework in improving patient outcomes. These studies will serve to empirically test the utility of the framework and provide clinical evidence supporting its application in regenerative medicine.

## Figures and Tables

**Table 1:** Sleep hygiene.

Improving the Quality of Sleep
Refrain from taking naps that exceed one hour in duration during the daytime
Establish a consistent bedtime and adhere to it every day
Maintain a regular waking time daily
Avoid engaging in intense exercise up to one hour before bedtime
Limit the time spent in bed unless for 2–3 days of rest
Steer clear of stimulating activities before entering the bedroom
Ensure a calm mental state before bedtime
Reserve the bed solely for sleeping, avoiding other activities
Sleep a comfortable bed
Keep the bedroom at a consistent and cool temperature
Power down light-emitting devices
Refrain from engaging in mentally demanding work before bedtime
Avoid excessive planning for the next day while lying down
Consider the 3-2-1 rule: eat up to 3 h before, exercise up to 2 h before, and limit screen usage up to 1 h before sleep

**Table 2:** Key biomarkers analyzed in blood check-ups.

Biomarker	Biological Relevance	Reference
Albumin	Maintains plasma oncotic pressure and plasma volume, transports hormones, vitamins, oligominerals and drugs, and exerts an antioxidant and anti-inflammatory role.	[58]
Zinc	Zinc deficiency is significantly associated with metabolic disorders. It has a critical effect on homeostasis, immune function, oxidative stress, apoptosis, and aging.	[59]
Vitamin C	Vitamin C is a water-soluble agent, serving as an antioxidant and playing a crucial role as a co-factor in the biosynthesis of collagen, as well as in the metabolism of carnitine and catecholamines. Additionally, it is essential for the absorption of dietary iron.	[60]
Vitamin D	Vitamin D, a fat-soluble vitamin, holds significance in maintaining calcium homeostasis and influencing bone metabolism. The addition of Vitamin D through supplementation has the potential to elevate testosterone levels, which in turn may improve lean muscle mass and reduce adipose tissue.	[61]
Ferritin	Ferritin plays a crucial role in maintaining iron homeostasis and is implicated in various physiological and pathological processes. In clinical practice, ferritin is primarily employed as a serum marker to assess total body iron stores.	[62]
Homocysteine	While this protein is pivotal for cellular homeostasis in humans, it also displays a valuable role as a biomarker for diseases. Elevated levels of plasma total homocysteine have been linked to conditions such as neural tube defects, impaired childhood cognition, macular degeneration, primary stroke, and cognitive impairment in the elderly.	[63]
TSH	Thyroid-stimulating hormone serves as the primary stimulant for the production of thyroid hormones by the thyroid gland. TSH prompts thyroid follicular cells to release triiodothyronine (T3) or thyroxine (T4) hormones.	[64]
T3 and T4 (T4 is a precursor to T3)	T3 and T4 regulate energy metabolism. They play a key role in energy production within cells, help regulate body temperature, affect cardiovascular function, contribute to the central nervous system’s development, and influence muscle function.	[65,66]
DHEA	Dehydroepiandrosterone stimulates the breakdown of triglycerides in adipose tissue, influences insulin signaling pathways, increases glucose uptake in adipocytes, and enhances insulin sensitivity in individuals with DHEA deficiency or abnormal glucose tolerance. Additionally, it plays a role in the inactivation of cortisol to cortisone within adipose tissue.	[67]
ALP	Alkaline phosphatase is an enzyme that plays a critical role in various metabolic processes, including bone mineralization, liver function, and nutrient absorption. Elevated levels of alkaline phosphatase in blood tests can indicate liver disease, bone disorders, or other conditions affecting metabolic health.	[68]

**Table 3:** SDIMMMER grading scale.

Domain	Questions/Assessment Criteria	Grading Scale (0–4)
Sleep	(1) How many hours do you sleep per night? A—1 to 2 (0 points) B—3 to 4 (1 point) C—5 to 6 (2 points) D—7 (3 points) E—8 (4 points) (2) How often do you wake up during the night? A—Never (4 points) B—Rarely (3 points) C—Sometimes (2 points) D—Often (1 point) E—Always (0 points) (3) How long (in minutes) did you usually take to fall asleep in the last month? A—0 to 15 min (4 points) B—16 to 30 min (3 points) C—31 to 45 min (2 points) D—46–60 min (1 point) E—More than 60 min (0 points) (4) How often do you wake up tired? A—Never (4 points) B—Rarely (3 points) C—Sometimes (2 points) D—Often (1 point) E—Always (0 points) (5) How often do you use electronic devices before going to sleep? A—Never (4 points) B—Rarely (3 points) C—Sometimes (2 points) D—Often (1 point) E—Always (0 points)	[ ] 0 [ ] 1 [ ] 2 [ ] 3 [ ] 4
Diet	(1) What types of foods do you typically eat? A—Mostly healthy foods (4 points) B—Predominantly healthy foods with occasional unhealthy choices. (3 points) C—Balanced mix of healthy and unhealthy foods (2 points) D—Mostly unhealthy foods with some healthy options. (1 point) E—Consistently poor food choices. (0 points) (2) How often do you consume fried foods? A—Once a month or never (4 points) B—Twice a month (3 points) C—3 times in a month (2 points) D—Once a week (1 point) E—Twice or more a week (0 points) (3) How often do you eat fruits and vegetables? A—Once a month or never (0 points) B—Twice a month (1 point) C—3 times in a month (2 points) D—Once a week (3 points) E—Twice or more a week (4 points) (4) How often do consume sugar? A—Once a month or never (4 points) B—Twice a month (3 points) C—3 times in a month (2 points) D—Once a week (1 point) E—Twice or more a week (0 points) (5) How often do you eat fast food or takeout meals? A—Once a month or never (4 points) B—Twice a month (3 points) C—3 times in a month (2 points) D—Once a week (1 point) E—Twice or more a week (0 points)	[ ] 0 [ ] 1 [ ] 2 [ ] 3 [ ] 4
Microbiome	(1) How often do you consume probiotic-rich foods? (i.e., Yogurt, kefir, sauerkraut, kimchi, miso, kombucha). A—Once a month or never (0 points) B—Twice a month (1 point) C—3 times in a month (2 points) D—Once a week (3 points) E—Twice or more a week (4 points) (2) How often do you consume fiber-rich foods? (i.e., Whole grains, nuts, seeds, quinoa, granola). A—Once a month or never (0 points) B—Twice a month (1 point) C—3 times a month (2 points) D—Once a week (3 points) E—Twice or more a week (4 points) (3) How often do you use antibiotics? A—Once a year or never (4 points) B—Twice a year (3 points) C—3 Times a year (2 points) D—4 times a year (1 point) E—More than 4 times a year (0 points) (4) How often do you experience bloating or gas after meals? A—Never (4 points) B—Rarely (3 points) C—Sometimes (2 points) D—Often (1 point) E—Always (0 points) (5) How often do you experience constipation or diarrhea? A—Never (4 points) B—Rarely (3 points) C—Sometimes (2 points) D—Often (1 point) E—Always (0 points)	[ ] 0 [ ] 1 [ ] 2 [ ] 3 [ ] 4
Metabolism	(1) Do you have metabolic problems? (i.e., Diabetes, high blood pressure, high cholesterol, obesity). A—No. (4 points) B—Yes, only one. (3 points) ________________________ C—Yes, two. (2 points) ____________________________ D—Yes, three. (1 point) _________________________________ E—Yes, more than three. (0 points) ___________________________ (2) How often do you feel excessively tired during the day? A—Never (4 points) B—Rarely (3 points) C—Sometimes (2 points) D—Often (1 point) E—Always (0 points) (3) Has your weight changed in recent years? A—Yes. I’ve gained weight and I’m overweight (0 points) B—Yes. I’ve fluctuated in weight (2 points) C—No. It has remained stable (4 points) (4) How often do you feel hungry between meals? A—Never (4 points) B—Rarely (3 points) C—Sometimes (2 points) D—Often (1 point) E—Always (0 points) (5) Do you have thyroid problems? (i.e., Hypo or hyperthyroidism). A—No (4 points) B—Yes, under control (2 points) C—Yes, not under control (0 points)	[ ] 0 [ ] 1 [ ] 2 [ ] 3 [ ] 4
Medications	(1) How often do you use Nonsteroidal anti-inflammatory drugs (NSAIDs) A—Never (4 points) B—Rarely (3 points) C—Sometimes (2 points) D—Often (1 point) E—Always (0 points) (2) How often do you use medications without a prescription or medical advice? A—Never (4 points) B—Rarely (3 points) C—Sometimes (2 points) D—Often (1 point) E—Always (0 points) (3) How often do you have side effects or interactions with the medications you take? A—Never (4 points) B—Rarely (3 points) C—Sometimes (2 points) D—Often (1 point) E—Always (0 points) (4) Do you use the following medications: anticoagulants, antidepressive, statins or proton pump inhibitors (i.e., omeprazole, esomeprazole and lansoprazole)? A—No. (4 points) B—Yes, only one. (3 points) ________________________ C—Yes, two. (2 points) ____________________________ D—Yes, three. (1 point) _________________________________ E—Yes, all of them. (0 points) (5) How would you rate your compliance with medication instructions? A—Poor (0 points) B—Fair (1 point) C—Good (2 points) D—Very good (3 points) E—Excellent (4 points)	[ ] 0 [ ] 1 [ ] 2 [ ] 3 [ ] 4
Exams	(1) How often do you have a physical examination? A—Twice a year (4 points) B—Once a year (3 points) C—Once every two years (2 points) D—Once every three years (1 point) E—Once more than every three years (0 points) (2) How regularly have you undergone screenings such as mammograms and pap smears (for women) or prostate-specific antigen (PSA, for men) tests? A—Twice a year (4 points) B—Once a year (3 points) C—Once every two years (2 points) D—Once every three years (1 point) E—Once more than every three years (0 points) (3) How regularly have you undergone bone density exams? A—Twice a year (4 points) B—Once a year (3 points) C—Once every two years (2 points) D—Once every three years (1 point) E—Once more than every three years (0 points) (4) How regularly have you undergone a complete blood count? A—Twice a year (4 points) B—Once a year (3 points) C—Once every two years (2 points) D—Once every three years (1 point) E—Once more than every three years (0 points) (5) How regularly have you undergone cardiovascular health assessments, including EKGs, stress tests, or echocardiograms? A—Twice a year (4 points) B—Once a year (3 points) C—Once every two years (2 points) D—Once every three years (1 point) E—Once more than every three years (0 points)	[ ] 0 [ ] 1 [ ] 2 [ ] 3 [ ] 4
Rehabilitation	(1) How often do you practice aerobic exercises? (i.e., walking, running or jogging, cycling, swimming, dancing) A—I don’t practice aerobic exercises (0 points) B—At least once a week (1 point) C—2 times a week (2 points) D—3 to 5 times a week (3 points) E—More than 5 times a week (4 points) (2) How often do you feel stressed or anxious? A—Once a month or never (4 points) B—Twice a month (3 points) C—3 times a month (2 points) D—Once a week (1 point) E—Twice or more a week (0 points) (3) How often do you practice stress management techniques? (i.e., Deep breathing exercises, mindfulness meditation, yoga, journaling, social support). A—Once a month or never (4 points) B—Twice a month (3 points) C—3 times a month (2 points) D—Once a week (1 point) E—Twice or more a week (0 points) (4) How would you rate your compliance with therapies of rehabilitation? A—Poor (0 points) B—Fair (1 point) C—Good (2 points) D—Very good (3 points) E—Excellent (4 points) (5) How often do you practice strength training? A—I don’t practice strength training (0 points) B—At least once a week (1 point) C—2 times a week (2 points) D—3 to 5 times a week (3 points) E—More than 5 times a week (4 points)	[ ] 0 [ ] 1 [ ] 2 [ ] 3 [ ] 4

## Data Availability

No new data were created or analyzed in this study. Data sharing is not applicable to this article.
